# Deposition of Bacteriorhodopsin Protein in a Purple Membrane Form on Nitrocellulose Membranes for Enhanced Photoelectric Response

**DOI:** 10.3390/s130100455

**Published:** 2012-12-27

**Authors:** Young Jun Kim, Pavel Neuzil, Chang-Hoon Nam, Martin Engelhard

**Affiliations:** 1 Laboratory of Nanomedicine, Korea Institute of Science and Technology Europe (KIST-Europe), Campus E 7.1, Saarbrücken, Germany; E-Mails: yjunkim@kist-europe.de (Y.J.K.); pavel@kist-europe.de (P.N.); 2 Max-Planck-Institut für Molekulare Physiologie, Otto-Hahn-Str. 11, 44227 Dortmund, Germany; E-Mail: martin.engelhard@mpi-dortmund.mpg.de; 3 Department of Life Science, Chonbuk National University, Jeon Ju 561-756, Korea

**Keywords:** bacteriorhodopsin (bR), purple membrane (PM), indium tin oxide (ITO), positively charged nitrocellulose membrane (PNM)

## Abstract

Bacteriorhodopsin protein (bR)-based systems are one of the simplest known biological energy converters. The robust chemical, thermal and electrochemical properties of bR have made it an attractive material for photoelectric devices. This study demonstrates the photoelectric response of a dry bR layer deposited on a nitrocellulose membrane with indium tin oxide (ITO) electrodes. Light-induced electrical current as well as potential and impedance changes of dried bR film were recorded as the function of illumination. We have also tested bR in solution and found that the electrical properties are strongly dependent on light intensity changing locally proton concentration and thus pH of the solution. Experimental data support the assumption that bR protein on a positively charged nitrocellulose membrane (PNM) can be used as highly sensitive photo- and pH detector. Here the bR layer facilitates proton translocation and acts as an ultrafast optoelectric signal transducer. It is therefore useful in applications related to bioelectronics, biosensors, bio-optics devices and current carrying junction devices.

## 1. Introduction

Four decades ago Bacteriorhodopsin protein (bR) was extracted from the membrane of *Halobacterium salinarum* found in high salt water depositions after its original discovery in 1967 [1,2]. The bR contains the chromophore retinal and this protein is known to be one of the simplest biological energy convertors [3]. bR has an excellent thermal, chemical, photochemical and photoelectrical stability, which makes bR an attractive material in devices for photosignal transduction. bR protein is also known as the purple membrane (PM) due to its purple color patches. The PM is composed of bR protein (75% wt.) and lipids (25% wt.) in a two dimensional (2D) hexagonal lattice of *Halobacterium salinarum*. PM occurs naturally in this 2D structure and it is essential for subsequent one directional proton pumping perpendicular to the lattice resulting in high efficiency. Due to bR stability, the PM retains its color and photochemical activity in dry conditions and can withstand temperatures above 100 °C [4]. Upon illumination with light at 570 nm, the bR molecule induces a *trans-cis* isomerisation of the retinal [5]. This process is followed by transitions of the retinal bound bR complex through a number of intermediate states to go back to the ground state bR. The intermediate states are quasi-stable and identified with letters: K, L, M, N, and O. All have different absorption spectra, different lifetimes in the range from milliseconds to picoseconds and different protonation states of the Schiff bases and amino acids [6,7]. The overall result includes the transfer of protons from the cytoplasmic side of cell membrane to the extracellular environment. It contributes to building up of the proton gradient across the membrane resulting in electrochemical potential and thus local pH changes [8]. This proton gradient provides the energy required for the *H. salinarium* cell activities by supporting adenosine triphosphate (ATP) synthesis from adenosine diphosphate (ADP) [9]. Orientation of the bR membrane is crucial for one-directional proton pumping and thus generation of maximum electrical potential [10]. Li *et al.* [11] have reported on poly-L-Lysine/bR two layer deposition and also provided a comprehensive list of other methods including Langmuir-Blodgett (LB) deposition [12], electrophoretic sedimentation [13], self-assembly [14], electrostatic layer by layer deposition [15], antigen-antibody molecular recognition [16], sol-gel encapsulation [17], use of polymer as immobilizing matrix [18], *etc.* Yamada *et al.* [19] recently described two other methods such as spin casting and dip coating techniques. In this study, positively charged nitrocellulose membrane (PNM) is used as substrate for the deposition of bR protein. Due to the negative charges of the cytoplamsic part of bR, bR proteins get aligned with the positive charge of the nitrocellulose membrane, thus during illumination protons move in the extracellular site direction. During the experiment, pH at the electrodes was modulated. Besides water-based experiments we have also used dry PM films. Absence of water assures that there is no washing off of bR from the PM. It also provides a typical light driven bR response by inducing electrical current between electrodes.

## 2. Experimental

### 2.1. Chemicals

All chemicals were purchased from Sigma (Schnelldorf, Germany) and Roth (Karlsruhe, Germany). Deionized water for washing and dilution was prepared by MilliQ (Millipore, Ltd. Darmstadt, Germany).

### 2.2. PM preparation

The PM was extracted from *Halobacterium salinarum* by method described earlier by Oesterhelt and Stoeknius [20]. Briefly, *H. salinarum* was cultured in a Petri dish containing 2% agar medium. It was kept illumined by yellow light at 45 °C on a reciprocal shaker at 200 rpm for 3 days. Bacterial cells were harvested and resuspended in basal salt solution (3 M NaCl) with 50 ng DNAase and 5 ng RNAase. The suspension was dialyzed against water for several hours to complete cell lysis. The solution was subsequently centrifuged at 50,000 rpm to remove the soluble proteins and partially digested polynucleotides. Membrane fragments were sediment into a purple coloured pellet. Fractionation of the membrane fragments was performed on linear sucrose gradients (25–50% sucrose with a 60% solution at the bottom of the tubes). Gradients were run for 20 hours at 25,000 rpm and later on purple membrane fraction was collected. It was then resuspended in 5 mM Tris-HCl buffer solution at pH 7.0. Concentration of PM was determined by measurement of absorption spectra at 568 nm with assumption that the PM extinction coefficient was 62,700 M^−1^·cm^−1^.

### 2.3. Fabrication of pH Electrodes

IrO_2_ films were electrochemically deposited on the Pt microelectrodes by a potential-cycling method in an 100 mL aqueous solution containing 0.15 g IrCl_4_ (99.5%), 2 mL H_2_O_2_ (30%) and 0.5 g (COOH)_2_·2H_2_O. We have calibrated the electrodes by measurement of a V_cell_ as a function of pH. We have used standard pH buffer solutions with pH value of 4, 7 and 11, respectively (see Figure 5). This method was adapted from previously described technique by Yamanaka [21]. The calibration process was repeated few times to demonstrated reversibility of the process as well as stability.

### 2.4. PM immobilization onto Nitrocellulose Membrane in Photovoltatic Cell

**Figure 1.**
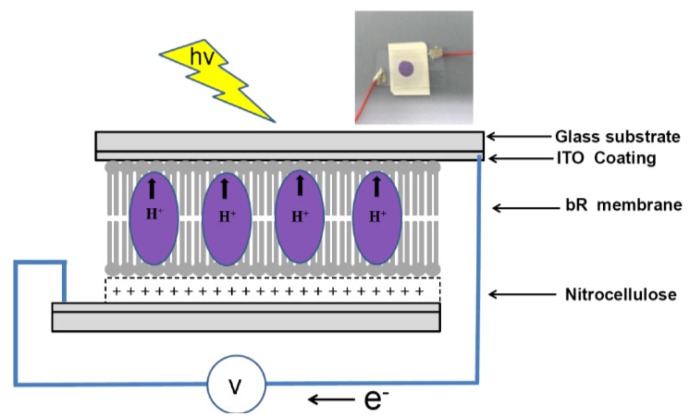
Schematic representation of measurement setup of oriented bR films on nitro cellulose photocell. A picture of the fabrication in the inset with 20 μL bR solution deposited on PNM.

The photovoltaic cell consists of two ITO coated electrodes with nitrocellulose film and PM sandwiched between them (Figure 1).

The cell was made by 1.5 cm^2^ nitrocellulose membrane (PNM) placed on top of a 2 cm^2^ ITO coated glass. Purified PM (20 μL, 1.2 mg/mL) was deposited on the top of the PNM and dried at room temperature. Both ITO electrodes were connected to electronic instruments the measure the PM/PNM properties.

### 2.5. Photoelectrochemical Measurements

Photocurrent, photovoltage and impedance measurements were done using a standard three electrode electrochemical cell configuration with saturated Ag/AgCl reference electrode. Photoelectro-chemical measurements of the assembled PM on PNM were conducted under illumination of 40, 60 and 100 mW/cm^2^, respectively. SCHOTT KL 200 (Galvoptics Ltd, Basildon, UK) equipped with built-in UV filter was used as the light source. We have used an Autolab PGSTAT-12 digital potentiostat/galvanostat, using GPES software (Eco Chemie BV, Utrecht, The Netherlands) for instrumentation.

## 3. Results and Discussions

When light first strikes the photoelectric device, there is a rapid instantaneous decrease in the potential. The light driven proton transfer was optimized and a minor loss of sensitivity was observed after one measurement of light exposure.

**Figure 2.**
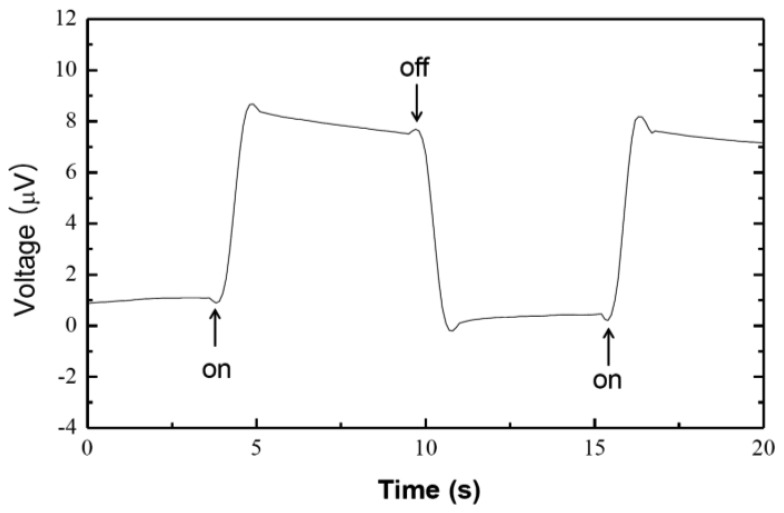
Photovoltage difference across the PM/PNM membrane under illumination with power of 100 mW/cm^2^.

**Figure 3.**
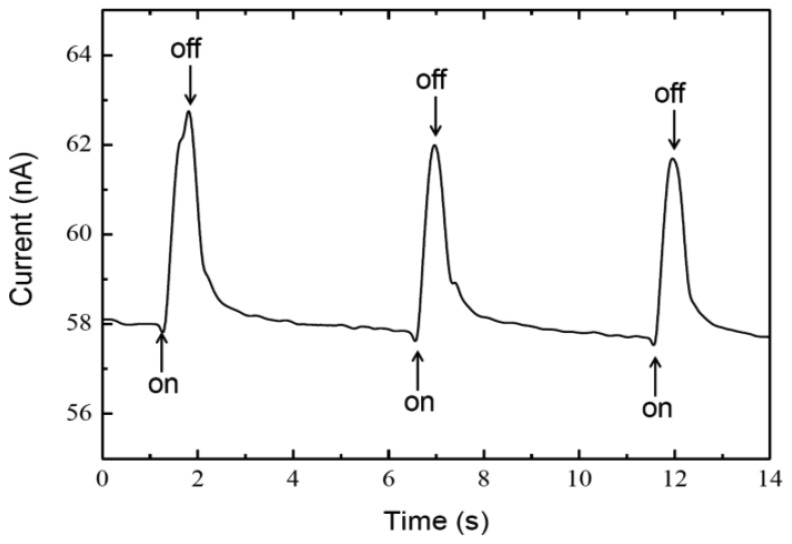
Photoinduced current from PM/PNM as a function of illumination time. The peak wavelength of incident light was 537 nm.

When a PM-based photocell was illuminated with 100 mW/cm^2^, photocurrent and photovoltage values of approximately 6.3 nA and 8 μV were detected, respectively (Figures 2 and 3). Reproducibility of the photosensor is comparable to that of previous reports [22–24]. Upon light exposure, upper side of the ITO acted as cathode (electron donor) confirming the orientation of the upper PM nitrocellulose whereas the bottom side acted as an anode (electron acceptor). The amplitude of photo induced current went back to its original value of about 18 nA in dark condition. This current is significantly smaller than the theoretical maximum of current. That is probably caused by PNM structure.

We have used a nitrocellulose substrate which is made of crisscrossed fiber compressed together. Also the top surface of the membrane is not in one plane and not flat either. Hence, there is practically more surface aligned with PM as monolayer. However, even if PM on the PNM achieves high-density monolayers, each of them have different orientations in the PNM surface. Finally, this could lower the power produced by PM.

**Figure 4.**
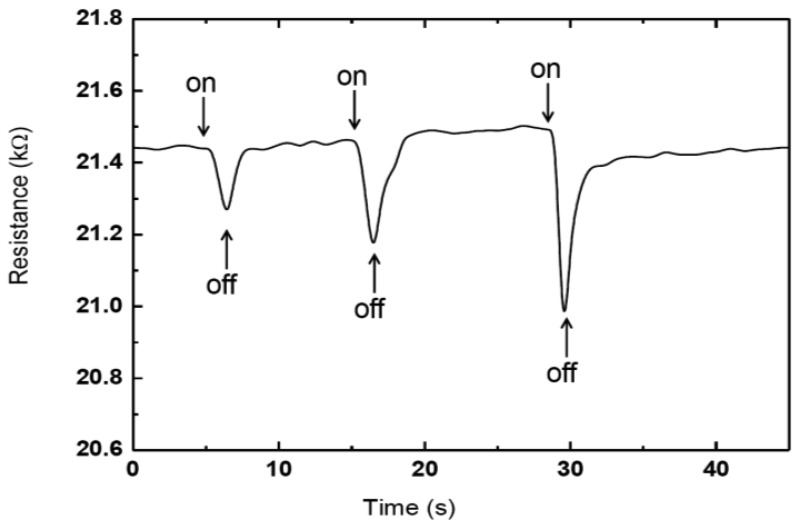
Impedance changes as a function of time at 1 kHz with 40, 60 and 100 mW/cm^2^, respectively.

A typical impedance measurement of a photocell is shown in Figure 4. Measurements were taken at a frequency of 1 kHz. The effect of incident light intensity on cell impendence was measured at 40, 60 and 100 mW/cm^2^, respectively. Decreases in impedance from 21.2 to 21.1 and 20.8 kΩ were observed as the intensity amplitude was increased from 40 to 60 and 100 mW/cm^2^, respectively.

**Figure 5.**
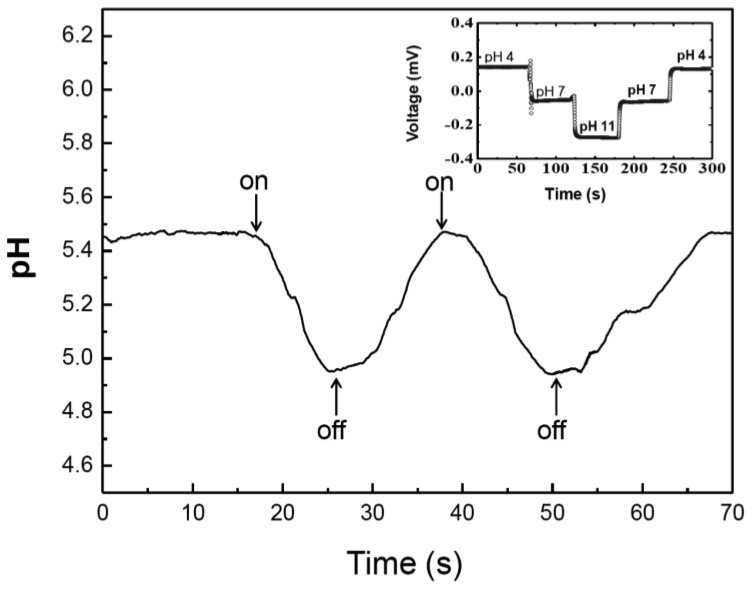
Changes in pH of PM as a function of light exposure with power of 100 mW/cm^2^. Calibration of the electrode system is shown in the inset.

As shown in Figure 5, Photovoltaic responses were measured at pH values of 4, 7 and 11, respectively. We have found that the cell voltage linearly depends on the pH. After the calibration, we have tested the PM/PNM-based cell in ddH_2_O. Figure 5 shows the pH values of PM calculated from the calibration curve with illumination of 100 mW/cm^2^. Once the cell was illuminated, pH dropped from its original value of 5.47 to 4.95. Once the light was switched off, pH value increased back to its original value. Under illumination protons are released leading to a decrease of pH value (anodic polarization). However, once the light is turned off reprotonation of the bR cytoplasmic side occurs resulting in an increase of pH value (cathodic polarization). This study showed that negatively charged cytoplasmic part of the bR in PM deposited on the PNM contributed to the one-directional proton pumping. Furthermore, PM deposited only on one side of the relatively thick PMN could explain the missing negative peak of a photocurrent [25]. We can assume that protons can be prevented from reaching the electron acceptor. It also showed that light responsive dry PM membrane on nitrocellulose produced an electrochemical signal. We have measured it as a local pH change. This result contributes to the proton translocation and not the electric charge displacement. Our results demonstrated that PM can be easily and rapidly immobilized on PNM to serve as a primary electron donor. The study focused on the light driven proton pump and revealed that PNM can be alternatively used for photoelectrical device as a protein coating material.

## 4. Conclusions

The device was based on a combination of optically transmitting and electrically powering systems. Deposition of PM on nitrocellulose is a simple technique leading to promising device development for pH monitoring. It can be used in conjunction with semiconducting or conducting biomaterial photosensors for signal amplification and direct transmission architecture. Furthermore, PNM can be integrated with conducting nanomaterials of low affinity into flexible membranes to ensure the sensitivity of the photoelectrical test.
